# Cancerous Tumor in the Right Parotid Region

**Published:** 1859-01

**Authors:** 


### ARTICLE VII.

*Cancerous Tumor in the Right Parotid Region.* Translated for the American Journal of Dental Science.

RAYMOND LOPEZ, laborer, born at Valmojado, Province of Toledo, sixty-six years of age, married, of sanguine temperament, and of a good constitution, appeared at our clinic on the 1st of June. The following is an account of his condition:

In the right parotid region at the angle of the jaw, was found a tumor of the size of a nut, (?) hard, indolent and movable; it was impossible to discover the extent of its adherance. We further remarked on the right side of the face, in the region of the malar bone, an ulcer about an inch in extent, irregular in shape, with thick, hard, callous and reversed edges; it was granulated and covered with unhealthy pus. The ulcer had appeared about four months before the patient came to our clinic, and had commenced in a varicose swelling on the right jaw, quite long, conical and without pain or inflammation. The patient had broken it twice, but he derived no advantage; the irritation on the contrary increased, and the tumor developed more fully. It occurred to us that we had better apply a ligature, but when the tied portion came away the tumor commenced to grow again.

The tumor in the parotid region had scarcely existed a month before the patient came to our clinic. We applied emollient cataplasms and different caustics, without obtaining the least amelioration. On the 9th we proceeded to extirpate the tumor in the following manner:—The first incision was made from the top to the bottom of the parotid region, from which flowed a thick liquid emanating from the source of the tumor; then we proceeded to the dissection of the skin, (integument?) which was easily accomplished, till the internal part was reached, where the adherance became intimate and deep. It was necessary to operate with much prudence and dexterity as much on account of the limited surface for operation as on account of the anatomical importance of the parts and the depth of the attachments: we feared also wounding an important vessel. This done, we hastened to circumscribe the ulcer with four incisions crossing each other, and forming a parallelogram, and then the affected portion was easily removed, always, however, taking care not to injure the facial artery which was very distinctly seen at the bottom of the wound. The lips of the wound were united linearally by three points of a twisted suture and three other interlaceal sutures, and lint was placed on the parotid region, which was kept there by proper bandages.

The patient returned on the 6th of July; the incisions were cicatrized, but with a puckering of the healthy tissues.*

* Thus it is evident, that, in trying to effect a linear reunion of a wound that has the form of a parallelogram, it is important to avoid, if possible, puckering of the tissues. We think that one is too disposed, at the present day, to have recourse in every case, to immediate reunion without calculating upon the results. Without speaking of the accidents which it particularly predisposes, such as erysipelas, &c., there are many cases where it should be rejected. We remember that a young, well instructed surgeon, who will leave behind him the most valuable and honorable recollections in the annals of science, after having removed a wen from the back, exactly reunited the lips of the semi-lunar incision which he had made. Dreading the influence of the atmospheric air, he thought, that after the suture, he ought to use collodion and sticking plaster. The fragment of skin, although so thin as to be transparent, did not mortify, the wound promising a speedy cicatrization; but, some days after the head surgeon of the hospital, when he came in, plunged the bistoury through this fragment of skin and into the sack which was under it. A quantity of pus escaped. The fever abated and the patient was able to sleep. In this case it was better, instead of a painful and difficult dissection, to remove the skin which covered the tumor. Dr. G.

